# Prize of the Alumni Association of the Chicago Medical College

**Published:** 1869-05-15

**Authors:** S. A. M. Williams

**Affiliations:** Sec’y and Treas’r, 166 State St., Chicago, Ill.


### EDITORIAL.

The publication of the CHICAGO MEDICAL JOURNAL having passed into the hands of the subscriber, all payments for subscription and accounts, either past or future, must be made to me, unless otherwise directed. Subscribers who have neglected to pay their subscriptions on volumes previous to the present year must remit at an early day or the publisher will be compelled to discontinue sending them the JOURNAL.

The editorial management will remain as heretofore, in the hands of Dr. J. Adams Allen, assisted by Dr. W. Hay, and to whom communications in his department should be addressed.

J. WOOLCOTT, HOOKER,
Room 12, Union Building.

CHICAGO, May 1st.

The following communication explains itself, and we take much pleasure in publishing to the professional world these evidences of *esprit de corps* and progress in its young members. Deprived of the fostering care extended to it by paternal governments in older civilizations, the development of the science and art of medicine devolves upon its own students, and the alumni of the Chicago Medical College have manifested a most praiseworthy spirit in putting forth, so early in their professional career, this effort to encourage each other in well doing. W. H.

### *Prize of the Alumni Association of the Chicago Medical College.*

This is a prize of one hundred dollars, to be awarded for the best essay on some medical or surgical topic. The subject of the essay is left to the option of the competitor.

The prize is open for competition to all the members of the Association good on the Secretary's books. Every alumnus of the Chicago Medical College is earnestly urged to become a member of the Association by sending his name and one dollar to the Secretary. The Committee on Competing Essays consist of N. S. Davis, M.D., E. Andrews, M.D., H. A. Johnson, M.D., and Walter Hay, M.D. Each essay must be designated by a device or motto, and must be accompanied by a sealed envelope bearing the same device or motto, and containing the name and address of the author. The envelope belonging to the successful essay will be opened, and the name of the author announced, at the regular annual meeting of the Association, also, at the annual Commencement of the College in March, 1870. The competing essay must be sent to the Secretary of the Association on or before the 15th Feb., 1870.

It is expected that the prize essay, and others of marked value, will be published in pamphlet form, and furnished to members of the Association.

S. A. M. WILLIAMS, Sec'y and Treas'r,
166 State St., Chicago, Ill.

